# Knowledge of nurses regarding dysphagia in patients post stroke in Namibia

**DOI:** 10.4102/curationis.v38i2.1564

**Published:** 2015-11-09

**Authors:** Anthea Rhoda, A. Pickel-Voight

**Affiliations:** 1 Faculty of Community and Health Sciences, University of the Western Cape, South Africa

## Abstract

**Background:**

Stroke patients commonly experience dysphagia post stroke. Complications of dysphagia include aspiration leading to chest infection and pneumonia, malnutrition, dehydration, and a subsequent increased risk of death. Its early diagnosis and management is an important prerequisite for recovery from stroke during the rehabilitation phase. As nurses are the first health personnel that interact with a patient post stroke, it is important that they are knowledgeable and skilled in the screening of these patients for dysphagia.

**Objective:**

The aim of the study was to determine the knowledge and factors associated with knowledge of nurses regarding dysphagia in stroke patients.

**Methods:**

The study used a quantitative survey to determine the knowledge of the nurses employed at an intermediate hospital in Namibia. A convenient sample of 182 participants completed a self-administered questionnaire with closed-ended questions, which was developed by the researcher. The data was analysed using descriptive and inferential statistics.

**Results:**

The findings of the study confirmed that nurses have a moderate knowledge of the signs, symptoms, and complications of dysphagia, but poor knowledge about its management. Training and experience in the care of dysphagia patients was a stronger predictor of knowledge than the initial qualification or years of experience as a nurse.

**Conclusion:**

Post basic training in dysphagia would better equip nurses to manage stroke patients in the acute phase.

## Introduction and rationale

Stroke is a common cause of death and disability worldwide (World Health Organisation [WHO] 2013). One of the most common impairments after the onset of stroke is neurogenic oro-pharyngeal dysphagia. Dysphagia is the inability to swallow or difficulty to hold food and fluid in the mouth (Hughes 2011:3; Nazarko 2010), and occurs in approximately 50% of stroke patients in the acute phase (Ickenstein *et al*. 2012:573). Dysphagia has been associated with a high incidence of respiratory complications, increased risk of aspiration pneumonia, malnutrition, dehydration, persistent disabilities, and mortality (Morris 2006:559; Ramsey, Smithard & Kalra 2005:218; Rofes *et al*. 2010:7).

Early diagnosis and effective management of dysphagia could minimise aspiration pneumonia and mortality (Hinchey *et al*. 2005:1975), and improve oral movements as well as nutritional status, leading to better post stroke outcomes (Jacobsson *et al*. 2000:263; Rosenvinge & Starke 2005:592). Dysphagia screening should take place in the first 24 hours after admission of stroke patients to hospital, to ensure that nutrition and hydration are well managed (Head *et al*. 2007; National Institute for Health and Clinical Excellence [NICE] 2008). The diagnosis of dysphagia is usually made by a speech and language therapist (SLT) (Martino, Pron & Diamant 2000:25). To screen for dysphagia, Hinchey *et al*. (2005:1975) recommended a simple bedside testing protocol for all stroke patients before they are given fluid or food. This test could be administered by trained personnel such as nurses if the SLT is not available. Improving the skills of nurses in the screening of dysphagia is therefore important (Cichero, Heaton & Bassett 2009:1657). A lack of resources such as limited budgets and staffing could, however, affect training of nurses to improve skills and knowledge. It has also been reported by Blackwell and Littlejohns (2010) that the care of patients after stroke in sub-Saharan Africa may not be adequate because of limited health care budgets, insufficient resources, and lack of qualified and knowledgeable health care professionals.

The continued focus by governments and local health care planners on poverty and other health priorities such as infectious diseases (Malaria and AIDS) has resulted in an under-appreciation of the current and future impact of non-communicable diseases, such as stroke, in Africa (Lemogoum, Dagaute & Bovet 2005:99). This could result in a lack of policy and action in training health care workers in essential routine tasks, such as performing a simple bedside swallow test, avoiding aspiration, and ensuring adequate hydration and nutrition in stroke patients (Kolapo & Vento 2011:708).

For that reason, training for the identification and management of stroke and its complications needs to be developed on the African continent on a progressively larger scale. Training of health care workers and home caregivers should include knowledge of how to administer a simple screening test, such as the bedside swallow test, as well as the correct positioning of the patient during mealtime (Kolapo & Vento 2011:708).

## Problem statement

Indisputable evidence reports improved outcomes for stroke patients managed by a multidisciplinary team in a stroke unit (Stroke Unit Trialists 2013:18). This multidisciplinary team consists of physiotherapists, occupational, SLTs, physicians and nurses. The management of dysphagia is primarily the role of the SLT. At the time of the study there was no SLTs employed at the Oshakati State Hospital. This could lead to the inadequate management of dysphagia of stroke patients admitted to this hospital. To address this situation nurses could assist with the screening of stroke patients with dysphagia. It should be noted that the screening and management of dysphagia by nurses does not replace other health professionals, but is an important step in the early detection of dysphagia and thus facilitates the targeted treatment of dysphagia (Barnard 2011). Nurses do however need to be knowledgeable with the condition to screen effectively. Furthermore, information about the level of knowledge regarding dysphagia amongst nurses could guide the implementing of suitable continuing professional development interventions in this area.

## Purpose of the study

The study therefore aimed to determine the factors associated with knowledge of dysphagia amongst nurses at an intermediate hospital in Namibia.

## Research design

A quantitative, descriptive, non-experimental survey was used to collect the data. The purpose of quantitative research is to describe, explain or predict phenomena. This is performed with the systematic collection of numerical data, and analysed with the help of statistical methods (Polit, Beck & Hungler 2001). In the current study, ‘the phenomenon’ refers to the determination of the level of knowledge of nurses regarding dysphagia.

### Population and sampling

The target population of the study consisted of 500 nurses who were employed at the intermediate state hospital of Oshakati. Of these 500 nurses, 254 were enrolled nurses (ENs) and 246 were registered nurses (RNs) (Kakungulu 2011). All 500 nurses were conveniently selected to participate in the study. Convenience sampling is a non-probability sampling technique where samples are selected because of their convenient accessibility (Polit *et al*. 2001).

## Data collection method

### Data collection instrument

No standardised questionnaire about the knowledge of dysphagia in stroke patients amongst nurses relevant to the research setting, and pertinent to the research question, was found in existing literature. The researcher therefore designed a self-administered questionnaire from literature (Cichero *et al*. 2009:1650; Werner 2010:E3), which was guided by the objectives of the study. The questionnaire contained closed-ended questions with several options as answers. The following options were offered: Disagree –− Unable to decide − Agree. Section A of the instrument included questions relating to socio-demographic characteristics of the study participants. The socio-demographic section asked for information pertaining to the respondent's age, position as a nurse, qualifications, years of experience in nursing, and experience with stroke. Section B included questions about the knowledge of the signs and symptoms of dysphagia, complications of dysphagia such as aspiration pneumonia, malnutrition, dehydration, or mortality of stroke patients, and the management of dysphagia in stroke patients.

To determine the knowledge of the participants about dysphagia, the responses were scored as follows: a correct response resulted in 1 point, an incorrect response resulted in 0 point, and an unsure response were counted as 0 point, as personally conveyed by C. Basuayi during August 2012. The scoring of 0 for an unsure response was based on the assumption that nurses who were ‘unsure’ were also ‘unaware’ of the correct response at the time of the data collection (Park *et al*. 2011). Each section was scored independently.

The points were then combined to obtain a total score for each section. As an example, section B1 scored a total of 13 points, section B2 a total of 10 points, and section B3 a total of 7 points. The total score of all sections was 30. The level of knowledge was classified as follows: a score of 75% and above was classified as ‘high’, from 50% to 74% was classified as ‘moderate’, and a score from 50% and below was classified as ‘low’ (personal communication with C. Basuayi during August 2012). This was applied for overall score as well for each section.

### Validity and reliability of the developed questionnaire

In order to ensure face and content validity of the questionnaire, it was given to five health profession experts (speech language therapists) with experience in the field. One is employed by the Ministry of Health and Social Services and working at Windhoek Central Hospital, two are working in a private practice in Windhoek, and two are working in a stroke unit in Germany. All the experts agreed that the questionnaire sufficiently covered all aspects of dysphagia. The test-retest method was used to determine reliability of the questionnaire. Fifteen nurses, from the same population, were recruited to participate and complete the questionnaires twice with a two-week interval between the first and second completion of the questionnaires. The nurses took 15 minutes to complete the questionnaire. The initial results from the test-retest yielded Intra Class Correlation (ICC) results of < 0.7. The questionnaire was adapted, and all questions with poor and moderate results from the questionnaire were removed. In section B1, *signs and symptoms of dysphagia*, the following eight questions were removed: eating fast, neck stiffness, coughing 20 min after a meal, neck swelling, toothache, difficulty in smelling, contracting herpes simplex, and severe headache. In section B2*, complications of dysphagia*, one question was removed, which was ‘delay of recovery’. In section B3, *management of dysphagia*, the following three questions were removed: ‘All stroke patients should be screened before being given food or drink for the first time, ‘There are not reliable tests for difficulty in eating and swallowing problems’, and ‘The best way to find out if the patient has eating or swallowing difficulties, is to ask the patient.’ After removing these questions, an ICC value of > 0.7 could be achieved. The data from these questionnaires were not included in the main study.

### Data collection procedure

Ethical clearance and permission was obtained for this study from the relevant authorities (project no 13/2/28). All nurses were invited to participate in the study by way of an invitation letter. The participants were recruited through invitations distributed throughout the hospital, either through personal invitation by the researcher, or by informing the supervisor of each ward, and creating an awareness of the study at nursing staff meetings. The nurses were given the permission to complete the survey questionnaire during a two-week period, between 11h00 and 14h00. For that purpose a dedicated room in the administration block of the hospital was provided and equipped with sufficient tables and chairs. The researcher first explained the purpose of the study to the participants and answered queries about the questionnaire. Each participant was then asked to sign an informed consent form which indicated that they had understood the purpose of the study, and their participation was voluntary. After completing the consent forms and the questionnaires, the nurses were asked to place the consent forms and the questionnaires in separate boxes. Afterwards, consent forms and questionnaires were locked separately in a cabinet to which only the researcher had access. Participant questionnaires were given identity codes to ensure anonymity.

### Data analysis

All questionnaires were numerically coded, captured, and analysed using the statistical package for social sciences (SPSS) 22.0 version. First descriptive statistics were used to describe the dependent variable (knowledge) and independent variables (socio-demographic characteristics of participants and experience with stroke). A correlation matrix was then computed to determine whether there were significant associations between the demographic variables and knowledge of the symptoms of dysphagia, or complications of dysphagia and management of dysphagia. Significant associations were tested at a 0.05 alpha level. Correlation was appropriate to determine the association between the stated variables as it is a mathematical index of the association between two sets of data (Pretorius 2007). Coefficients of determination were calculated to determine the percentage of the variance on a particular variable that was explained as a function of knowledge about another variable (Balnaves & Caputi 2001).

Regression analyses were performed to test the predictive relationship between identified predictor variables and knowledge of the symptoms, complications, and management of dysphagia respectively. The ensuing multiple regression analyses tested three models for significance. Model 1 regressed position at work, qualifications, and training caring for patients with eating problems onto knowledge of the symptoms of dysphagia. The second model regressed position at work, qualifications, experience caring for a patient with dysphagia, and training in caring for patients with eating problems onto knowledge of the complications of dysphagia. The third model regressed position at work, qualifications, experience caring for a patient with dysphagia, training in caring for patients with eating problems, knowledge of the symptoms of dysphagia and knowledge of the complications of dysphagia onto knowledge of complications of dysphagia.

## Results

### Response

Of the 500 nurses employed at the hospital 188 nurses accepted the invitation, completed and returned the questionnaires. Four questionnaires were excluded from the study, because more than 10% of the questions were not completed. Therefore, the final sample consists of 184 questionnaires.

### Demographic characteristics of participants

The age of the study sample ranged from 21 to 66 years with a mean age of 43.88 years (SD = 13.12). Further results are presented in Table 1.

**TABLE 1:** Socio-demographic characteristics of participants.

Demographic variables	Characteristics	Frequency *N*	Percentage
Age (years) (*n* = 182)	21–30	48	26.4
	31–40	21	11.5
	41–50	54	29.7
	51–60	46	25.3
	61 and above	9	4.9
Position (*n* = 184)	Registered nurse	103	56.0
	Enrolled nurse	81	44.0
Qualification (*n* = 183)	Certificate	81	44.0
	Diploma	61	33.2
	Bachelor of Science	39	21.2
	Master of Science	2	1.1
Years of experiences (*n* = 183)	0–3	30	16.3
	4–6	27	14.7
	7–9	7	3.8
	10 and above	119	64.7
Ward of practice (*n* = 184)	Medical	39	21.1
	Orthopaedic	13	7.1
	Surgery	21	11.4
	Outpatient department	9	4.9
	Maternity	12	6.5
	Gynaecology	9	4.9
	Psychiatric	9	4.9
	Paediatric	23	12.5
	TB-Ward	10	5.4
	Ophthalmology	7	3.8
	ICU	8	4.3
	Operating theatre	8	4.3
	X-ray	1	0.5
	Casualty	8	4.3
	Administration	7	3.8

### Participants’ work experience with management of stroke patients

The majority (*n* = 153/184, 83.2%) of the participants reported that they had cared for stroke patients, and 119/184 (64.7%) stated that they had cared for stroke patients with eating or swallowing difficulties. With regard to training in eating or swallowing difficulties, it was found that the majority of the participants 134/184 (71.2%) did not receive any formal training. Further, 134/184 (72.8%) nurses indicated that they were not satisfied with their knowledge regarding eating and swallowing difficulties, and 93.5% (*n* = 172/184) of nurses stated that they would like to have further formal training.

### Knowledge of nurses regarding dysphagia

The correct response rate for all three knowledge sub-sections (sign and symptoms, complication and management of dysphagia) combined was 57.34%. This results in an average of 17.65 points out of 30 points which could be achieved and demonstrates a moderate overall knowledge level.

The participants’ knowledge of the signs and symptoms, complications and management dysphagia, are presented in Tables 2, 3 and 4. The level of knowledge was ranked. A score of 75% and above was classified as ‘high, 50% to 74% was classified as ‘moderate’, and a score of 50% and below was classified as ‘low’.

**TABLE 2:** Participants’ knowledge about the signs and symptoms (*n* = 184).

Variables	Disagree *n* (%)	Unable to decide *n* (%)	Agree *n* (%)	Total score per variable
Coughing whilst eating.	20 (10.9)	12 (6.5)	152 (82.6)*	1
Skin irritations.	128 (69.6)*	34 (18.5)	22 (12)	1
Feeling of food getting stuck in the throat.	8 (4.3)	7 (3.8)	169 (91.8)*	1
Choking on saliva during non-mealtimes.	38 (20.7)	36 (19.6)	110 (59.8)*	1
Poor movement of the tongue.	17 (9.2)	17 (9.2)	150 (81.5)*	1
Food remains in the mouth.	17 (9.2)	9 (4.9)	158 (85.9)*	1
Poor chewing.	25 (13.6)	24 (13)	135 (73.4)*	1
Patients always cough if they aspirate.	17 (9.2)*	19 (10.3)	148 (80.4)	1
Difficulty closing lips.	58 (31.5)	31 (16.8)	95 (51.6)*	1
Weight loss.	53 (28.8)	17 (9.2)	114 (62)*	1
Frequent throat clearing after swallowing.	50 (27.2)	25 (13.6)	109 (59.2)*	1
Hoarse voice.	28 (15.2)	24 (13.0)	132 (71.7)*	1
Chest pain.	88 (47.8)	48 (26.0)	48 (26.1)*	1
**Total of score**	**-**	**-**	**-**	**13**

*, correct answer

**TABLE 3:** Participants’ responses on their knowledge of the complications (*n* = 184).

Variables	Disagree *n* (%)	Unable to decide *n* (%)	Agree *n* (%)	Total score per variable
Increased mortality	48 (25)	27 (14.7)	111 (60.3)*	1
Pneumonia	58 (31.5)	30 (16.3)	96 (52.2)*	1
Anaphylactic shock	65 (35.3)*	29 (15.8)	90 (48.9)	1
General weakness	22 (12.0)	10 (5.4)	152 (82.6)*	1
Problems with digestion	27 (14.7)*	28 (15.2)	128 (69.6)	1
Aspiration	16 (8.7)	4 (2.2)	164 (89.1)*	1
Dehydration	18 (9.8)	7 (3.8)	159 (86.4)*	1
Sudden heart attack	48 (26.1)*	50 (27.2)	86 (46.7)	1
Malnutrition	24 (13.0)	2 (1.1)	158 (85.9)*	1
Haematemesis (vomiting blood)	90 (48.9)*	49 (26.6)	45 (24.5)	1
**Total of score**	**-**	**-**	**-**	**10**

*, correct answer

**TABLE 4:** Participants’ responses on their knowledge of management (*n* = 184).

Variables	Disagree *n* (%)	Unable to decide *n* (%)	Agree *n* (%)	Total score per variable
Patients with a feeding tube need daily oral hygiene (mouth washing and brushing of the teeth).	9 (4.9)	6 (3.3)	169 (91.8)*	1
Thickened liquid should be avoided.	34 (18.5)*	13 (7.1)	138 (74.5)	1
Watery liquids are the safest substances to drink.	9 (4.9)*	4 (2.2)	171 (92.9)	1
All patients with difficulty in swallowing need a feeding tube.	46 (25)*	13 (7.1)	125 (67.9)	1
The best position whilst feeding the patient is when the patient lies flat on his back.	133 (72.3)*	15 (8.2)	36 (19.6)	1
The patient can always eat normal hospital food.	146 (79.3)*	13 (7.1)	25 (13.6)	1
Feeding tube is only indicated in patients with impaired consciousness.	97 (52.7)*	19 (10.3)	68 (37)	1
**Total of scores**	**-**	**-**	**-**	**7**

*, correct answer

### Participants’ knowledge of signs and symptoms of dysphagia

For this section, a total score of 13 points could be achieved. The average correct response score was 8.4 points (64.62%). The achieved overall percentage of 64.62% indicates a moderate knowledge. Scores for individual items are presented in Table 2.

### Participants’ knowledge of the complications of dysphagia

For the section relating to knowledge of complications, a total score of 10 points could be achieved. The overall achieved percentage of 58.15% indicated a moderate knowledge. Scores for individual items are presented in Table 3.

### Patients’ knowledge of the management of dysphagia

As shown in Table 4, for this section a total score of 7 points could be achieved. The average correct response score was 3.45 points. The attained percentage of 49.28% indicates a poor knowledge on this area.

### Factors associated with knowledge of dysphagia

When controlling for other factors such as the qualifications and position of the nurse practitioner, experience in having provided care for a patient with eating problems, and knowledge of the symptoms and complications of dysphagia, training in caring for a patient with dysphagia remained a significant predictor of the management of dysphagia (Table 5).

**TABLE 5:** Regression analysis of variables regressed onto participants’ knowledge of dysphagia.

Model	Predictors	Outcome	*R*^2^	*b*
1	Training in caring for patient.	Knowledge of symptoms	.043	−.022
	Position.			−.298
	Qualifications.			−.128
	Caring for pt (excluded).			
2	Training in caring for patient.	Knowledge of complications	.070	−.148
	Position.			.026
	Qualifications.			.044
	Cared for patient with eating problem			−.184
3	Training in caring for patient.	Knowledge of management	.073	168
	Position.			−.169
	Qualifications.			.095
	Cared for patient with eating problem.			−.012
	Knowledge of symptoms.			.003
	Knowledge of complications.			.002

## Discussion

The aim of the study was to determine the nurses’ knowledge of the management of stroke patients. In the current sample, a total of 119.4/184 (64.7%) stated that they had cared for stroke patients with eating or swallowing difficulties. This is quite a large number, thus highlighting the need for these nurses to be able to manage this condition, because they could be the first health professionals the patient encountered on admission to hospital. Early identification of dysphagia and start of intervention can help sustain nutrition intake and reduce further complications (Bouziana & Tziomalos 2011:4). Knowledge in this area of management, therefore, becomes essential.

### Knowledge of signs and symptoms

The participants had moderate knowledge of the signs and symptoms of dysphagia. Participants agreed with 80.4% (*n* = 148) that ‘patients always cough if they aspirate’, which is an incorrect answer; only 9.2% (*n* = 17) of the participants answered this question correctly. Patients who have impaired sensation silent aspiration could occur without a cough or clearing of the throat (Masiero *et al*. 2008). According to Masiero *et al*. (2008), 64.2% of stroke patients presented aspiration, and of these, 20.8% aspirated silently. If silent aspiration is not diagnosed because it does not show any clear signs of aspiration pneumonia, the result can be an increase in morbidity and mortality (Leder & Suiter 2014). This result would be consistent with the results of Robertson (2008:129) and Smith (2006), where caregivers and nurses showed less awareness of the signs and symptoms of silent aspiration when compared to knowledge of the other signs and symptoms.

### Knowledge of complications

Only (52.2%, *n* = 96/184) correctly identified pneumonia as a complication in stroke patients suffering dysphagia. Robertson (2008) found a similar result in her study, where the participants were less aware about recurrent pneumonia amongst dysphagia patients. Aspiration pneumonia is a widespread complication amongst dysphagia patients, and it is associated with significant morbidity and mortality. Nurses need to be aware of the possibility of complication pneumonia caused by aspiration, and therefore it is important for them to identify dysphagia and to manage it correctly (Eisenstadt 2010). The participants also had a moderate level of knowledge about the complications of dysphagia. The moderate result in the question about mortality (60.3%, *n* = 111), indicates that nurses are aware of the complication of mortality amongst stroke patients. The mortality rate amongst stroke patients with pneumonia was six times higher than for those without pneumonia (Katzan *et al*. 2003). The researcher hypothesises that participants really considered the complication of mortality as being directly related to damage caused by the stroke and not as a result of pneumonia or other symptoms of dysphagia. This point has to be examined more closely in future investigations.

### Knowledge of management

The results revealed that the participants had poor knowledge of the management of dysphagia. It is known that early and effective treatment can decrease complications and subsequent death (Martino *et al*. 2000:2). The majority of the participants (92.9%, *n* = 171) agreed with the statement that ‘Watery liquids are the safest substances to drink’. In contrast, the literature recommends avoiding thin liquids, because many patients with dysphagia are unable to drink thin liquids safely, and therefore have a higher risk for penetration and aspiration of thin liquid whilst swallowing (Ney *et al*. 2009:410). The majority of participants, 74.5% (*n* = 138) agreed that ‘Thickened liquid should be avoided’. This is also incorrect as thickeners increase the viscosity of liquids. Using thickener decreases the rate of flow through the mouth and pharynx, and allows patients more time to initiate airway protection, thereby decreasing the risk of aspiration (Brady 2008:45; Cichero *et al*. 2009:1656; Clavé *et al*. 2006:1392). Rosenvinge and Starke (2005) investigated the persistent lack of compliance of nursing staff with the SLT recommendations for the use of thickeners for feeding dysphagia patients. Their findings show that 38% of nurses did not comply with SLT recommendations for thickening food for dysphagia patients. This shows a lack of awareness amongst a large proportion of nurses of the potentially harmful consequences of their handling. Ineffective management of patients with dysphagia could lead to aspiration pneumonia and subsequent death (Hinchey *et al*. 2005:1975).

### Association between demographic factors and participants’ knowledge of dysphagia

The results of the study indicated that training in the care of a patient with dysphagia was significantly linked to knowledge of the management of dysphagia. Various studies conducted by Ilott *et al*. (2013:1362) in England, Cichero *et al*. (2009:1657) in Australia and Colodny (2001) in the USA have confirmed that training can significantly improve knowledge of dysphagia. In addition, Harper (2007) has reported that nurses with more experience have a statistically significant higher knowledge score than nurses with less experience. This is confirmed by the recent study, which shows that not only nurses with training in caring for stroke patients show a higher level of knowledge of complications, but that experience of caring for stroke patients is also an important factor regarding knowledge of complications.

## Study limitations

The current study was only carried out in one hospital in Namibia, therefore, the results may not exactly reflect the knowledge of all nurses in this country.

The results of the study can also not be generalised to the nursing profession because the study has used a convenience sampling method with a relatively small sample.

## Recommendations

The authors suggest that a training programme specifically related to the management of dysphagia in stroke patients be implemented to assist the nurses in the management of stroke patients.

## Conclusion

The results of the present study showed that participants have a moderate knowledge regarding the signs and symptoms, and complications of dysphagia, but a poor knowledge about its management. It also established that nurses with further training have a higher knowledge score than other nurses without further training, regardless of the main educational background of the nurse. Therefore, it is important that nurses be trained in the basic principles of dysphagia management.
